# The Biomechanical Effect of the Sensomotor Insole on a Pediatric Intoeing Gait

**DOI:** 10.5402/2012/396718

**Published:** 2012-10-10

**Authors:** Akiyoshi Mabuchi, Hiroshi Kitoh, Masato Inoue, Mitsuhiko Hayashi, Naoki Ishiguro, Nobuharu Suzuki

**Affiliations:** ^1^Department of Orthopaedic Surgery, Nagoya University Graduate School of Medicine, 65 Tsurumai, Showa-ku, Nagoya, Aichi 466-8550, Japan; ^2^Department of Electrical Engineering and Bioscience, School of Advanced Science and Engineering, Waseda University, 3-4-1 Okubo, Shinjyuku-ku, Tokyo 169-8555, Japan; ^3^Semui College, Tokai College of Medical Science, 2-7-2 Meiekiminami, Nakamura-ku, Nagoya, Aichi 450-0003, Japan; ^4^The Institute for Developmental Research, Aichi Human Service Center, 713-8 Kagiya-cho, Kasugai, Aichi 480-0392, Japan

## Abstract

*Background*. The sensomotor insole (SMI) has clinically been shown to be successful in treating an intoeing gait. We investigated the biomechanical effect of SMI on a pediatric intoeing gait by using three-dimensional gait analysis. *Methods*. Six patients with congenital clubfeet and four patients with idiopathic intoeing gait were included. There were five boys and five girls with the average age at testing of 5.6 years. The torsional profile of the lower limb was assessed clinically. Three-dimensional gait analysis was performed in the same shoes with and without SMI. *Results*. All clubfeet patients exhibited metatarsal adductus, while excessive femoral anteversion and/or internal tibial torsion was found in patients with idiopathic intoeing gait. SMI showed significant decreased internal rotation of the proximal femur in terminal swing phase and loading response phase. The internal rotation of the tibia was significantly smaller in mid stance phase and terminal stance phase by SMI. In addition, SMI significantly increased the walking speed and the step length. *Conclusions*. SMI improved abnormal gait patterns of pediatric intoeing gait by decreasing femoral internal rotation through the end of the swing phase and the beginning of the stance phase and by decreasing tibial internal rotation during the stance phase.

## 1. Introduction

Gait disorders have become the most prevalent orthopedic problems in children. One of the most common complaints of the gait disorders in infants and children is an intoeing gait, which is caused by excessive anteversion of the proximal femur, internal tibial torsion, and/or metatarsal adductus [1–5]. A careful evaluation of the children is essential to rule out serious pathological conditions such as cerebral palsy, infantile Blount's disease, metabolic bone diseases, and skeletal dysplasias. An intoeing gait in the majority of patients without these pathological conditions is a minor problem and is observed to spontaneously improve with time [2]. The condition, however, sometimes produces functional problems such as frequent tripping. The parents or grandparents are concerned that the child will have a permanent disability or that the condition will interfere with the child's physical performance; thus they sometimes hope for some treatments [5]. A shoe modification with wedges or arch supports is one of the tolerable nonsurgical treatments for children to correct an intoeing gait and modify the gait pattern.

The sensomotor insole (SMI), which had been introduced by the German shoemaker Jarhling, was originally developed to improve abnormal gait patterns in children with spastic gait [6, 7]. It was expected to change the muscle tone of the lower limbs by stimulating the proprioceptors of the sole. Jahrling and Rockenfeller demonstrated improvement of the gait patterns in cerebral palsy patients with flexible equinus or equinovarus deformities using SMI [6]. This information prompted us to try the use of SMI for a pediatric intoeing gait, since the patients usually have more flexible feet than the cerebral palsy patients. In the present study, the effects of SMI on a pediatric intoeing gait were evaluated using the three-dimensional (3D) gait analysis with a Vicon motion capture system.

## 2. Materials and Methods

The parents of patients treated with SMI for their intoeing gait at our institution between January 2009 and December 2010 were invited to have their child participate in this prospective study, which was approved by an institutional review board. Patients who had rigid deformities of the feet with an ankle range of motion less than 30 degrees or forefoot abduction less than 10 degrees were excluded from this study. Patients who underwent gait analysis with the use of other systems were also excluded. Some patients could not complete testing because of the child's uncooperative behavior. Overall, 10 patients were included in this series (Table 1). There were five boys and five girls, and the average age at testing was 5.6 years (range, 3–9 years). Bilateral intoeing gait was observed in eight, right side in one, and left side in one. Among six congenital clubfeet patients who had initially been treated with serial casting and braces, two patients were additionally treated with extensive soft tissue releases. There were four patients in an idiopathic intoeing gait. The torsional profile was assessed with the patient prone on the examining table, as described by Staheli et al., so that the examiner can determine the amount of internal and external rotation of the hip as an indication of the amount of femoral anteversion, measure the thigh-foot angle in order to estimate tibial torsion, and examine the shape of the lateral border of the foot to assess the presence of metatarsal adductus [2].

**Figure 1:**
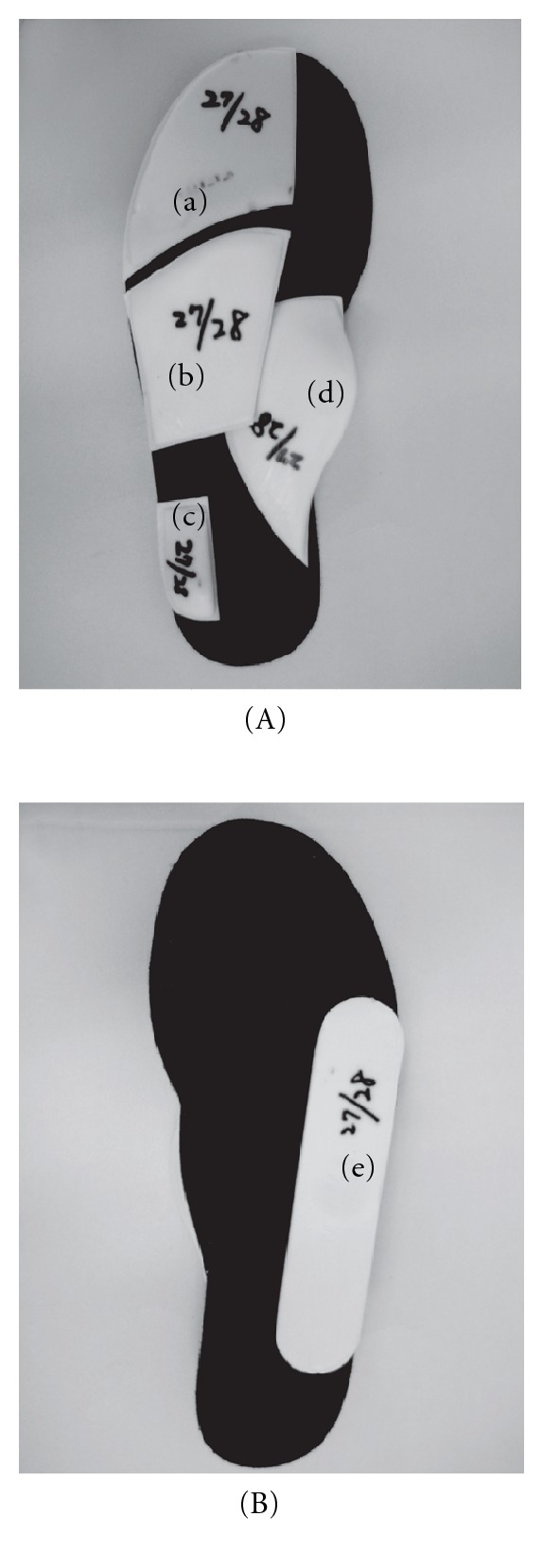
The upper (A) and the reverse (B) views of the sensomotor insole used for this study. It is consisted of five bars including a toe bar (a), a retro bar (b), a lateral heel bar (c), a medial heel bar (d), and a last bar (e).

SMI comprises of five exclusive bars, including medial and lateral heel bars, a retro bar, a toe bar, and a lateral wedge (Figure 1). Before first application of the insole, patients were individually ground to size Velcro trial elements that were attached to a base sole by the orthopaedic shoe technician. The trial elements can be readjusted repeatedly until the desired gait pattern has been achieved. Once the final position of the bars has been determined, the individual SMI was made up in the workshop using the care set [8].

The computerized motion analysis tests were performed with a six-camera VICON 3D motion system (Vicon MX system, Oxford, UK) during walking at a self-selected speed along a five-meter walkway using 16 passive retroreflective markers attached to specific bony landmarks of the lower extremities and the pelvis. Patients underwent gait analysis in the same shoes with and without SMI. Walking speed, stride length, and cadences were calculated using the Vicon Plug-in Gait (Vicon MX system, Oxford, UK), and the 3D joint kinematic data were obtained at 100 Hz for the hips, knees and ankles in the sagittal plane and the hips and knees in the coronal and transverse planes from bilateral lower extremities. Comparisons in walking speed, step length, cadences, and joint kinematic data of the lower extremities were made between the conditions with and without SMI. The data were analyzed using the VICON NEXUS Plug-in Gait and the custom-made software; the VICON Normalizer ver. 1.54. Gait cycle events that were defined by Perry were adopted for descriptions in this study [9].

Paired *t*-test was conducted to determine any difference among the demographics of the two groups (with and without SMI). The level of significance was set at *P* < 0.05. Analysis was performed with SPSS version 16.0 (SPSS Inc, Chicago, IL, USA).

## 3. Results

All patients with clubfeet showed residual metatarsal adductus without apparent hindfoot deformities. In four patients with idiopathic intoeing gait, excessive anteversion of the proximal femur was seen in three, and internal tibial torsion was found in two (one of the four patients had both deformities) (Table 1). Improvement of the gait pattern was clinically recognized after a couple of steps on wearing SMI in most patients.

**Figure 2:**
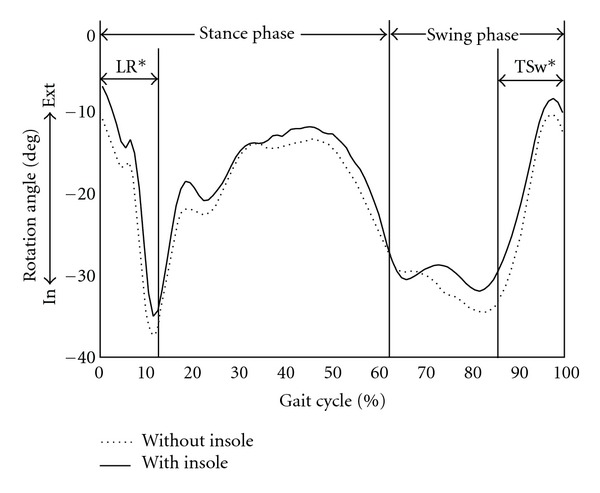
Average femoral rotation in transverse plane for patients with the sensomotor insole (solid) and without the insole (dotted). Positive values represent external rotation and negative values represent internal rotation. The patients with the sensomotor insole group demonstrated an increased external rotation of the proximal femur relative to the pelvis from the end of the swing phase to the beginning of the stance phase. LR: loading response, TSw: terminal swing, In: internal rotation, Ext: external rotation, *statistically significant.

**Figure 3:**
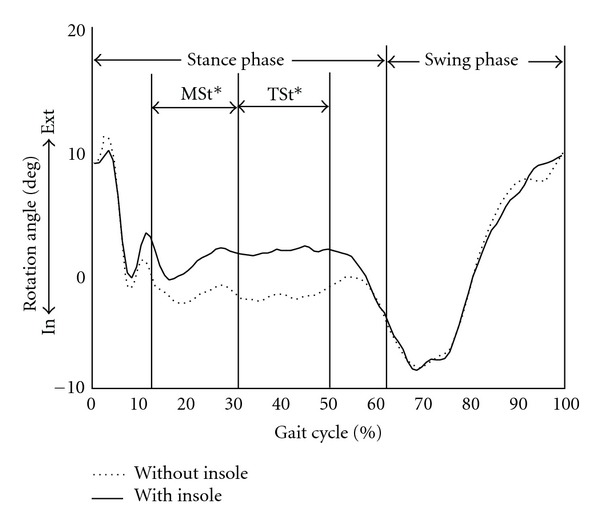
Average tibial rotation in transverse plane for the patients with the sensomotor insole (solid) and without the insole (dotted). Positive values represent external rotation and negative values represent internal rotation. The patients with the sensomotor insole group demonstrated an increased external rotation of the tibia relative to the femur in the middle of stance phase. MSt: mid stance, TSt: terminal stance, In: internal rotation, Ext: external rotation, *statistically significant.

There were significant differences in transverse plane motions of the hip and knee joints between the patients with and without SMI. SMI decreased internal rotation of the proximal femur relative to the pelvis in loading response phase (−18.3° ± 28.1° versus −21.6° ± 28.0°, *P* = 0.009) and terminal swing phase (−16.3° ± 27.4° versus −19.0° ± 26.4°, *P* = 0.047) (Table 2, Figure 2). SMI also decreased internal rotation of the tibia relative to the femur in mid stance phase (0.7° ± 12.5° versus −2.0° ± 14.9°, *P* = 0.030) and terminal stance phase (1.4° ± 11.9° versus −2.3° ± 14.5°, *P* = 0.042) (Table 3, Figure 3). There were no significant differences of the hip and knee kinematics in the sagittal plane between the patients with and without SMI. Ankle dorsiflexion was increased in loading response phase and terminal swing phase and decreased in terminal stance phase and terminal swing phase on wearing SMI. In regard to cadence parameters, SMI significantly increased the walking speed (67.9 m/min versus 64.9 m/min, *P* < 0.001) and the stride length (500 mm versus 477 mm, *P* < 0.001), although cadence did not differ between the two groups (137.6 steps/min versus 136.7 steps/min, *P* = 0.89) (Table 4).

## 4. Discussion

Li and Leong emphasized the importance of a correct diagnosis and understanding the causes and natural course of the condition in the management of a pediatric intoeing gait [10]. Thackeray and Beeson pointed out that identification of the level of torsional deformities (excessive femoral anteversion, internal tibial torsion, or metatarsal adductus) that produced an intoeing gait should be essential for planning of the specific intervention [3]. The present study, however, demonstrated that SMI improved abnormal gait patterns not only children with idiopathic intoeing gait but also those with congenital clubfeet, who had deformities of different levels in lower limbs. Although SMI cannot correct dynamic and structural abnormalities of the lower extremities of feet and its long-term effectiveness is unknown, it is one of the easy and effective treatment options in the management of a pediatric intoeing gait.

**Figure 4:**
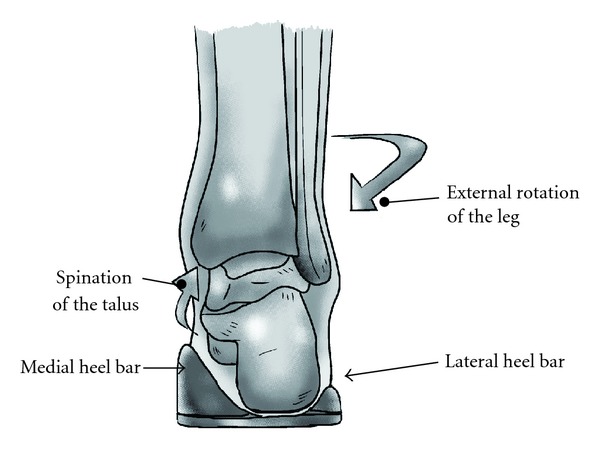
Hypothetical role of the medial and the lateral heel bar (rear view). The calcaneous kept in neutral position between the medial and the lateral heel bar suppresses pronation of the subtalar joint, leading to reduction of the internal rotation of the leg.

SMI inhibited internal rotation of the leg in loading response phase, which is usually observed in normal gait [11]. Nakajima reported that the foot progression angle, which is the angle formed by the direction of gait progression and the foot axis at mid stance, was reduced by using a lateral wedged sole, but it recovered in the use of a lateral wedge and an arch support that resembles SMI [12]. The present study revealed that the decreased internal rotation of the lower limb by SMI was shown to be due to decreased internal rotation of the proximal femur through the end of the swing phase and the beginning of the stance phase and decreased internal rotation of the tibia during the mid and terminal stance phases. Although the precise biomechanical effect of this specific insole on the hip and knee joints remains unknown, it is recognized that the leg-ankle-foot alignment is affected by the varus-valgus of the calcaneus [11]. We speculated that the calcaneus stabilized in neutral position between the medial and the lateral heel bar at the initial contact may provide favorable effects on entire lower limbs by suppressing pronation of the subtalar joint which is accompanied by internal rotation of the leg (Figure 4).

In addition to changing the rotational profile of the lower limb, SMI accelerated children's walking speed and increased stride lengths. The increase of the walking speed depended on the increase of the stride length because cadence did not show significant differences between children with and without SMI. SMI increased external rotation of the femur relative to the pelvis during the end of the swing phase, which reflected the internal rotation of the pelvis relative to the femur. The increment of stride lengths could be due to internally rotated pelvis toward the direction of progression in walking [13].

There are several limitations to this study. Young children were tested under a nonthreatening environment with parents present and distractions such as toys available in the present study, but the likelihood of not walking in their normal fashion while undergoing instrumented gait analysis is questionable. The foot progression angle, which is the most important variable to be measured, was not addressed in the present study because we unfortunately did not possess a force plate instrument that is indispensable to show the accurate direction of gait progression. The measurements of children without SMI may not reflect the actual gait situation because the shoes, which were adjusted on wearing SMI, would be a little large when children put them on without SMI. Finally, the number of patients examined in this study was limited because of difficulties in performing the gait analysis for toddlers or naughty children.

In conclusion, SMI improved an intoeing gait pattern in patients with congenital clubfeet and idiopathic intoeing by externally rotating the proximal femur through the end of the swing phase and the beginning of the stance phases and externally rotating the tibia during the mid and terminal stance phase, resulting in acceleration of the walking speed and increase in the stride lengths.

## Figures and Tables

**Table 1:** Summary of the patients.

Patients	Age (years)	Sex	Laterality	Diagnosis	Deformities
1	3	F	Bilateral	Clubfoot	Metatarsal adductus
2	7	M	Bilateral	Clubfoot	Metatarsal adductus
3	9	M	Bilateral	Clubfoot	Metatarsal adductus
4	5	F	Bilateral	Idiopathic intoeing gait	Excessive femoral anteversion
5	6	M	Bilateral	Clubfoot	Metatarsal adductus
6	7	F	Bilateral	Idiopathic intoeing gait	Excessive femoral anteversion and increased tibial internal torsion
7	3	M	Right side	Clubfoot	Metatarsal adductus
8	7	F	Bilateral	Idiopathic intoeing gait	Increased tibial internal torsion
9	4	F	Bilateral	Idiopathic intoeing gait	Excessive femoral anteversion
10	5	M	Left side	Clubfoot	Metatarsal adductus

*F: female, M: male.

**Table 2:** Femoral rotation in transverse plane.

Gait cycle	With the SMI (*n* = 18)	Without the SMI (*n* = 18)	*P* value
Loading response	−18.3° ± 28.1°	−21.6° ± 28.0°	0.009*
Mid stance	−20.5° ± 30.0°	−22.5° ± 31.0°	0.076
Terminal stance	−12.4° ± 28.0°	−13.6° ± 30.7°	0.275
Preswing	−17.7° ± 28.9°	−19.8° ± 30.0°	0.051
Initial swing	−29.0° ± 27.7°	−29.8° ± 30.0°	0.635
Mid swing	−30.3° ± 26.8°	−33.4° ± 26.9°	0.059
Terminal swing	−16.3° ± 27.4°	−19.0° ± 26.4°	0.047*

SMI: sensomotor insole.

^∗^Statistically significant.

±: External rotation/internal rotation.

**Table 3:** Tibial rotation in transverse plane.

Gait cycle	With the SMI (*n* = 18)	Without the SMI (*n* = 18)	*P* value
Loading response	4.6° ± 15.9°	4.2° ± 16.0°	0.767
Mid stance	0.7° ± 12.5°	−2.0° ± 14.9°	0.030*
Terminal stance	1.4° ± 11.9°	−2.3° ± 14.5°	0.042*
Preswing	−0.2° ± 10.7°	−1.6° ± 12.8°	0.317
Initial swing	−7.0° ± 14.0°	−7.0° ± 15.7°	0.882
Mid swing	−1.2° ± 15.8°	−0.8° ± 17.4°	0.752
Terminal swing	7.4° ± 13.6°	7.4° ± 14.7°	0.909

SMI: sensomotor insole.

^∗^Statistically significant.

±: External rotation/internal rotation.

**Table 4:** Gait parameter.

	With the SMI (*n* = 18)	Without the SMI (*n* = 18)	*P* value
Walking speed (m/min)	67.9 ± 9.4	64.9 ± 11.5	<0.001*
Step length (mm)	500 ± 81	477 ± 85	<0.001*
Cadence (steps/min)	137.6 ± 16.0	136.7 ± 14.9	0.89

SMI: sensomotor insole

^∗^Statistically significant.
